# TriFabs—Trivalent IgG-Shaped Bispecific Antibody Derivatives: Design, Generation, Characterization and Application for Targeted Payload Delivery

**DOI:** 10.3390/ijms161126037

**Published:** 2015-11-17

**Authors:** Klaus Mayer, Anna-Lena Baumann, Michael Grote, Stefan Seeber, Hubert Kettenberger, Sebastian Breuer, Tobias Killian, Wolfgang Schäfer, Ulrich Brinkmann

**Affiliations:** Roche Pharma Research & Early Development, Large Molecule Research, Roche Innovation Center Penzberg, D82377 Penzberg, Germany; klaus.mayer.km1@roche.com (K.M.); annalenabaumann@web.de (A.-L.B.); michael.grote@roche.com (M.G.); stefan.seeber@roche.com (S.S.); hubert.kettenberger@roche.com (H.K.); sebastian.breuer@roche.com (S.B.); tobias.killian@roche.com (T.K.); wolfgangschaefer@onlinehome.de (W.S.)

**Keywords:** knob-into-hole, disulfide stabilization, payload delivery, imaging, LeY, GPC3, CD33, saporin

## Abstract

TriFabs are IgG-shaped bispecific antibodies (bsAbs) composed of two regular Fab arms fused via flexible linker peptides to one asymmetric third Fab-sized binding module. This third module replaces the IgG Fc region and is composed of the variable region of the heavy chain (VH) fused to CH3 with “knob”-mutations, and the variable region of the light chain (VL) fused to CH3 with matching “holes”. The hinge region does not contain disulfides to facilitate antigen access to the third binding site. To compensate for the loss of hinge-disulfides between heavy chains, CH3 knob-hole heterodimers are linked by S354C-Y349C disulphides, and VH and VL of the stem region may be linked via VH44C-VL100C disulphides. TriFabs which bind one antigen bivalent in the same manner as IgGs and the second antigen monovalent “in between” these Fabs can be applied to simultaneously engage two antigens, or for targeted delivery of small and large (fluorescent or cytotoxic) payloads.

## 1. Introduction

Many different types and formats of bispecific antibodies (bsAbs) have been generated over the past years. These combine specificities of two antibodies in one molecule and enable binding of different epitopes or antigens [1,2]. BsAb formats include large Fc-containing molecules [3,4,5] as well as small entities, composed of two or more variable or even smaller binding domains fused to each other [6,7]. A large variety of bsAb formats were designed so far because different formats are required to address different therapeutic profiles. Factors that affect the choice and composition of bsAb formats include binding geometry and orientation of binding modules to each other (target accessibility, crosslinking), valences (avidity effects) and size (distribution and PK). In addition to that, robustness, stability, and manufacturing aspects are important points to consider for the development of bsAbs. This work describes the design, generation, and characterization of a novel IgG-shaped bispecific trivalent TriFab with novel composition and binding region geometry. Functionality of TriFabs is demonstrated by their ability to simultaneously bind to two antigens, and by applying TriFabs for bsAb-mediated targeted delivery of fluorophores or toxins to tumor cells.

## 2. Results and Discussion

### 2.1. Design and Generation of TriFabs

**Figure 1:**
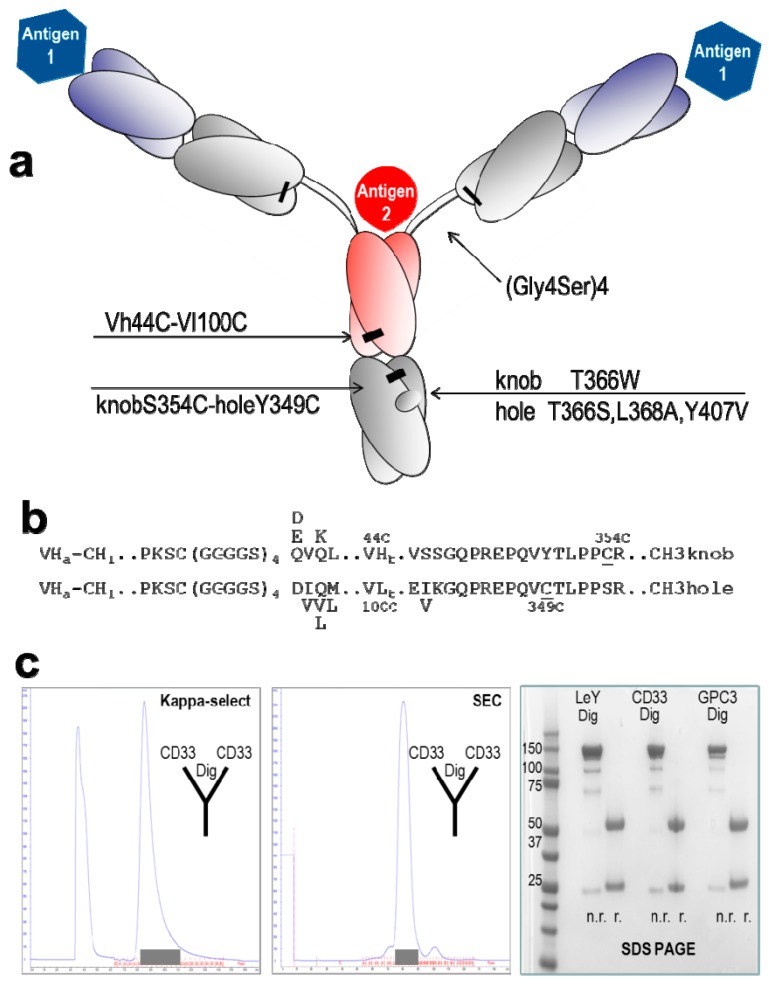
Design and generation of TriFabs. (**a**) TriFabs have the IgG hinge replaced by linker peptides without disulfides, and the CH2 regions by VH or VL. Hetero-dimerization is achieved by disulphide-stabilized knob-into-hole CH3, and by introducing a H44-L100 disulphide in the Fv. Interchain disulfides that connect light and heavy chains and the engineered stem heterodimer are indicated by black bars; (**b**) Fusion sequences linking CH1 with VH or VL with CH3. The N-terminus of Dig-VH and GPC3-VH is QVQL, DVQL for LeY-VH, EVQL for CD33-VH. The N-terminus of Dig-VL is DIQM, GPC3-VL DVVM, LeY-VL DVLM and CD33-VL DIQL. The N-terminal elbow region of CH3 hole is EIKG for GPC3, LeY and Dig, and EVKG for CD33; (**c**) TriFabs are purified from cell culture supernatants by affinity chromatography with kappa-select (**left** panel, Protein A does not capture our TriFabs). After loading supernatants to the column (left peak in Figure 1c), TriFabs were eluted with 100 mM Glycine-buffer (pH 2.5), subsequently adjusted to pH 6.0–7.5 with 1 M Tris (pH 9.0). This is followed by size exclusion chromatography (**middle** panel). Shaded boxes indicate fractions containing properly folded TriFab. The composition and purity of TriFabs obtained by this simple two-step procedure is shown in the SDS PAGE without (n.r.) and with (r.) sample reduction (**right** panel). The purification profiles are exemplarily shown for TriFabs with CD33-CD33-Dig specificity. The purification and profiles of other TriFabs are described in the suppl. data section.

The composition of TriFabs and the designed linker regions that connect the individual binding modules are shown in Figure 1a: two regular Fab arms are fused via flexible linker peptides to an asymmetric Fab-like entity which replaces the IgG Fc. This entity, which we term “stem region”, is composed of VH fused to CH3 with “knob”-mutations, and VL fused to CH3 with matching “holes”. The hinge region linker peptides that connect to the Fab arms do not contain interchain disulfides. This facilitates antigen access to the third binding site. To compensate the loss of hinge-disulfides between the heavy chains, the CH3 knob-hole heterodimer (T366W + T366S, L368A, Y407V according to the Kabat numbering scheme [8]) is linked by additional S354C-Y349C disulphides (Figure 1b) [7,9]. In addition, variable region of the heavy chain (VH) and variable region of the light chain (VL) of the stem region can be linked via additional (H44-L100) interchain disulphides [10]. This disulphide stabilizes the correct H-chain heterodimer, but it is not mandatory for heterodimerization to generate functional molecules: CH3 knob-hole interactions by themselves already provide sufficient heterodimerization, and the VH and VL domains that are also part of the stem region provide additional contributions.

A comprehensive description of the design including all fusion points and deviations from normal IgG sequences are provided in Figure 1. TriFabs were designed that address cell surface antigens—LeY, CD33, GPC3—and simultaneously bind digoxigenin or biotin- (hapten-)coupled payloads [11,12,13,14,15]. These TriFabs were produced transiently in HEK293 cells by co-transfection of three plasmids for CMV-promoter driven expression [4] of the three protein chains that together in a 2 + 1 + 1 ratio comprise TriFabs. These components are two light chains, one VH-CH3knob and one VL-CH3hole chain (Experimental Section). TriFabs become secreted into culture supernatants in the same manner as IgGs, indicating that hinge- and CH2 replacement does not compromise the folding and assembly process [16] of these bsAbs. We observed that TriFabs do not bind to Protein A (see Figure S1c for experimental details) because effective protein A capture of IgG involves the CH2 domain at the CH2-CH3 interface which is deleted in TriFabs. Purification is therefore achieved by protein-L followed by size exclusion chromatography. This generates TriFabs with yields of 3–20 mg/L (average 8 mg/L without process optimization, supplemental data). Due to the combination of the strong dimerizer domain CH3 [17] with four asymmetric hetero-dimerization modules (VH-VL + knob-holes + 2 interchain disulfides), purified TriFab preparations contain only desired knob-hole heterodimers without detectable amounts of wrongly assembled homo-dimers.

### 2.2. Stability of TriFabs

A problem that is frequently observed for a variety of engineered antibody derivatives is protein instability. To assess stability of TriFabs, we measured temperature-induced aggregation and unfolding by light scattering and tryptophan fluorescence, respectively (details in the Experimental Section and supplemental data). To evaluate stability of the format (independent of the specific binding regions), temperature-induced aggregation and unfolding was assessed for TriFabs that bind different cell surface antigens (CD33, LeY, GPC3) as well as different haptens (Bio, Dig). The results of these analyses (Table 1 and supplemental Figure S2) reveal that TriFabs are rather stable molecules with aggregation onset temperatures between 51 and 61 °C and denaturation temperatures between 58 and 66 °C for all TriFabs that were analysed (CD33-Dig, LeY-Dig, GPC3-Dig, CD33-Bio, LeY-Bio, GPC3-Bio). These temperature stability values are in the range of typical antibodies [18,19,20].

**Table 1:** Thermal stability of of TriFabs. Temperature-induced aggregation and unfolding of various TriFabs (hapten-specificity in the stem-Fv) was measured by light scattering and tryptophan fluorescence (details in M&M and supplemental data, Figure S2). Listed are aggregation onset temperatures (Tagg) defined as the temperature at which the scattered light intensity begins to increase, and denaturation temperatures (Tm) defined as inflection points of curves that represent ratios of fluorescence intensities at 350 and 330 nm.

TriFab	Tagg (°C)	Tm (°C)
CD33-CD33-Bio	57	58
CD33-CD33-Dig	51	66
GPC3-GPC3-Bio	56	58
GPC3-GPC3-Dig	61	65
LeY-LeY-Bio	52	59
LeY-LeY-Dig	60	66

### 2.3. TriFabs Retain the Binding Properties of Two Antibodies

**Figure 2:**
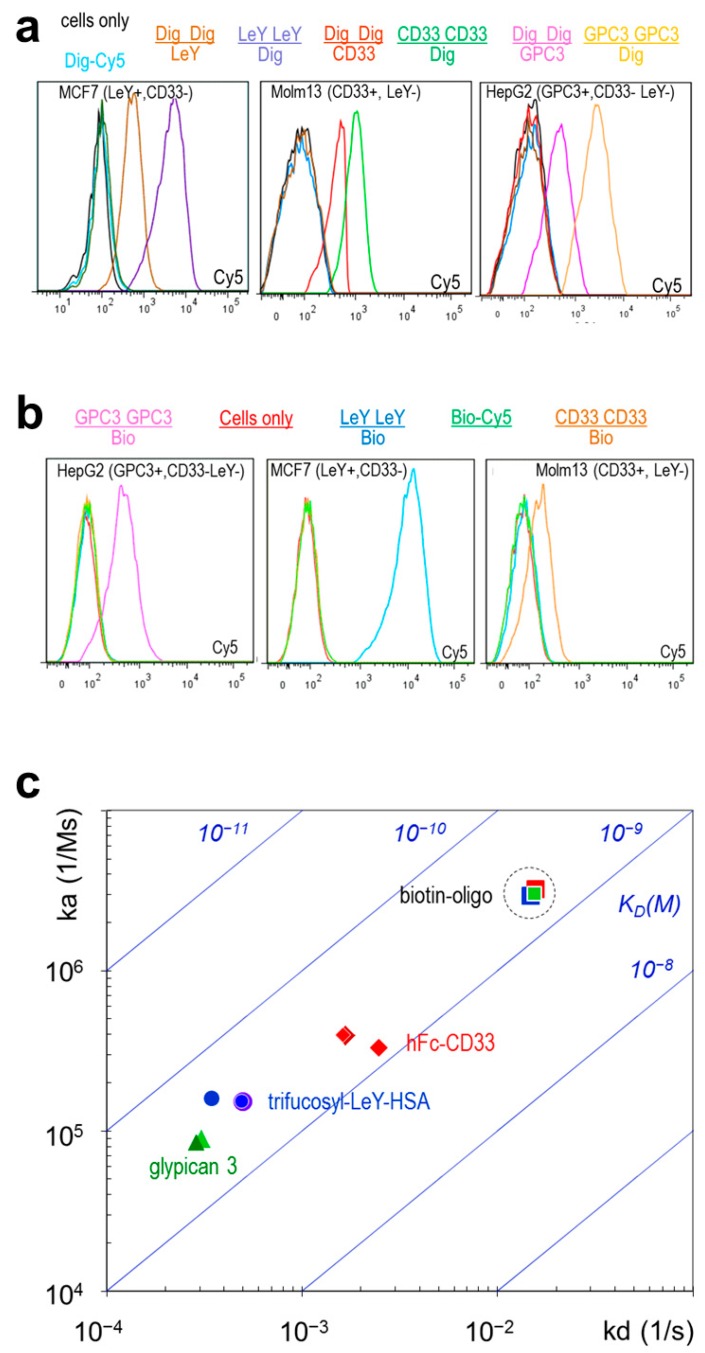
TriFab mediated targeted delivery of a small molecule. (**a**) TriFabs specific for glypican3 (GPC3, [12]), CD33 or LeY [11] combined with Dig-specificity were tested by FACS on LeY+,CD33− MCF7, CD33+,LeY− MOLM13 and GPC3+,Ley−,CD33− HepG2 cells with Dig-Cy5 payload [11]. “+” indicates expression of the listed antigen “−“indicates lack of expression. The binding specificities of the Fab arms are represented for each analysed molecule as “numerator” and the specificity of the Fv in the stem region as “denominator” with matched colour (except for Dig-Cy5 only or cells only which are light blue or black, respectively). Specific cell surface and hapten-binding is observed for TriFabs that bind cells with Fab arms and hapten in the stem region. Specific cell surface and hapten-binding is also observed for TriFabs that bind hapten bivalent with Fab arms and CD33 or GPC3 or LeY monovalent in the stem region; (**b**) TriFabs that have the Dig-binding moiety replaced by Biotin-binding moieties show same functionality when coupled to the payload Bio-Cy5 [13,14]; (**c**) Comparison of the SPR-determined affinities of Biotin-binding TriFabs which bind cell surface antigens with their Fab arms (bivalent) and Biotin (monovalent) with their stem-Fv. Listed are the on (ka) and off rates (kd) on *y*- and *x*-axes, respectively, as well as the resulting KD values (diagonal panels). Dashed circle: the Bio-binding of the stem region remains unaltered irrespective of which target antigen is addressed by the TriFab.

TriFabs access one antigen by their two Fab arms with the same affinity, orientation, and the same bivalent manner as regular IgGs. Surface resonance (SPR) analyses confirm that the two Fab arms of TriFabs bind antigen in the same manner as Fab arms of IgGs from which they were derived (Table 2). The second antigen is bound by the variable region of the “stem region” (as defined above), which is flanked by the Fabs. This Fv binds with the same affinity to digoxigeninylated payloads (antigen is a small hapten, payloads are oligonucleotides or fluorophores), or in one case specific but with reduced affinity to another biotinylated payload (a biotinylated oligonucleotide). The interspersed Fv also bind carbohydrate and protein antigens such as LeY, CD33 or GPC3 with the same specificity and (as shown for the CD33 antigen) with the same affinity as monovalent binding entities (Fabs) of their corresponding parent antibodies. Table 2 and Figure 2c summarize the results of surface plasmon resonance (SPR) analyses of the TriFabs with three different cell surface target specificities: The bivalent Fab arms of TriFabs bind antigen in the same manner as parent antibodies. The monovalent stem Fv (exemplarily shown for CD33 antigen, Biotin and Digoxigenin) has monovalent affinity (equivalent to a monovalent Fab fragment in case of CD33). Binding efficacy of the Fv that is part of the stem region (VH/VL-CH3) to cell surfaces depends on avidity, epitope accessibility and potential steric hindrance (which may explain the reduced affinity of biotin binders). Cell surface antigens CD33, GPC3 or LeY are accessible to Fv in the stem region in a monovalent manner and generate lower cell associated signals via fluorescence-activated cell sorting (FACS) analyses compared to bivalent binding (Figure 2).

**Table 2:** Antigen binding properties of TriFabs. Surface plasmon resonance (Biacore) measurements were applied to compare the affinities of TriFabs with those of their parent IgGs (see Figure 2c). Applied antigens were mono-biotinylated or mono-digoxigeninylated oligonucleotides, CD33Fc, LeY-BSA or recombinant GPC3 as previously described. * Data have been previously described [11,12,15]. Because the CD33 antigen is a (dimeric) Fc-fusion protein, monovalent binding of the reference molecule was determined with a monovalent Fab to avoid avidity effects.

Format	SPR	LeY Arm	GPC3 Arm	CD33 Arm	CD33 Stem	Dig Stem	Bio Stem
IgG	ka (1/Ms)	1.5 × 10^5^	8.5 × 10^4^	3.9 × 10^5^	1.9 × 10^5^ (Fab)	6.2 × 10^5^ *	2.0 × 10^7^ *
	Kd (1/s)	5.0 × 10^−4^	2.9 × 10^−4^	1.7 × 10^−3^	6.4 × 10^−3^ (Fab)	9.8 × 10^−3^ × *	1.0 × 10^−2^ *
	KD (M)	3.3 × 10^−9^	3.4 × 10^−9^	4.3 × 10^−9^	3.4 × 10^−8^ (Fab)	1.6 × 10^−8^ × *	6.2 × 10^−10^ *
TriFab	ka (1/Ms)	1.5 × 10^5^	8.6 × 10^4^	4.0 × 10^5^	2.4 × 10^5^	5.3 × 10^5^	2.9 × 10^6^
	Kd (1/s)	4.9 × 10^−4^	2.9 × 10^−4^	1.6 × 10^−3^	7.5 × 10^−3^	5.2 × 10^−3^	1.5 × 10^−2^
	KD (M)	3.2 × 10^−9^	3.4 × 10^−9^	4.1 × 10^−9^	3.1 × 10^−8^	9.8 × 10^−9^	5.1 × 10^−9^

### 2.4. TriFabs Enable Tumor Targeted Payload Delivery of Small Compounds

TriFabs that bind cell surface antigens as well as haptens were generated to evaluate TriFab-mediated payload delivery. Specific delivery of small compounds was demonstrated by FACS analyses of cells that were simultaneously exposed to digoxigeninylated fluorophores (Dig-Cy5, [11]), and to TriFabs that bind cell surface antigens and digoxigenin. Figure 2a shows that TriFabs deliver the small fluorescent compounds only to cells that express the cognate antigen on their surface: LeY-Dig delivers Dig-Cy5 to LeY-expressing MCF7 cells but not to LeY negative HEPG2 or Molm13 cells. Glypican-3 (GPC3) binding TriFabs deliver specifically to HEPG2 and CD33-binding TriFabs specifically to CD33 expressing Molm13 cells. Cell surface binding efficacy of TriFabs depends on valences and/or geometry of their cell surface binding arms. TriFabs that have their cell surface binding functionalities in bivalent Fab arms have higher Cy5-signals than cells that become targeted with TriFabs that bind to cells via their monovalent Fv in the stem region. Targeted delivery of small compounds is not restricted to TriFabs that bind to digoxigenin and Digoxigenin-containing payloads but works also for TriFabs that bind different haptens. Figure 2b shows that biotin-binding TriFabs can be applied in the same manner to deliver biotinylated payloads.

### 2.5. TriFabs Enable Tumor Targeted Payload Delivery of Protein Toxins

**Figure 3:**
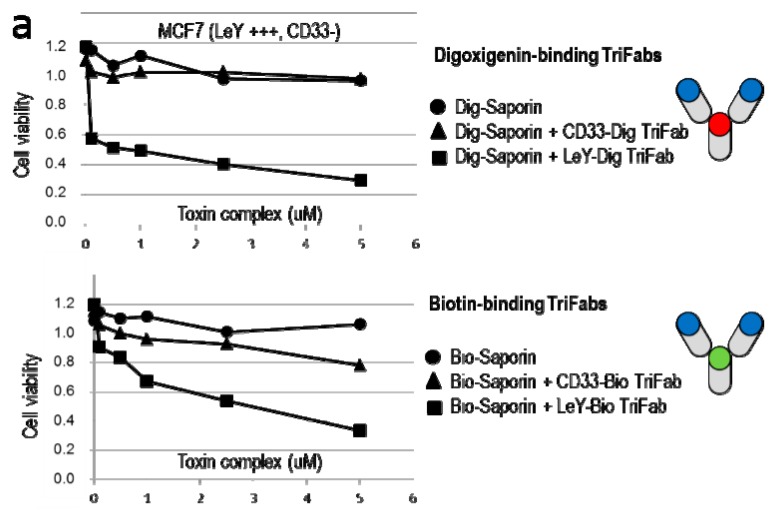

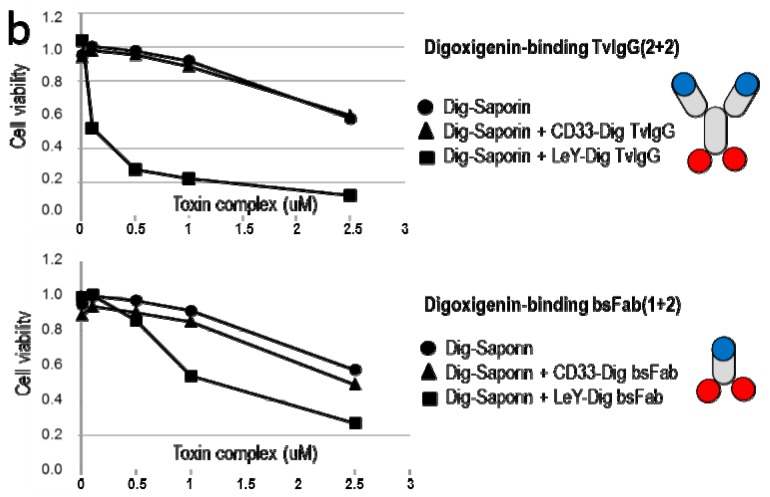
TriFab mediated targeted delivery of a large molecule. The applied bsAb formats are schematically depicted on the right (cell targeting entities in blue, Dig-binding entities in red and Bio-binding entities in green colour). (**a**) TriFab specific for Dig and GPC3 or CD33 or LeY combined with Dig-Saporin or Bio and GPC3 or CD33 or LeY combined with Bio-Saporin were applied for targeted delivery of saporin. TriFab-Saporin complexes were generated by a simple and robust charging procedure as previously described for hapten-coupled payloads [11,13,14,15]: Dig-Saporin and TriFabs are incubated in a 1:1 molar ratio in cell culture medium for at least 15 min, followed by subsequent dilution to the concentrations indicated. BrdU incorporation and ATP-content (Cell Titer Glo, CTG) assays were applied to measure the viability of cells 48 h after exposure to TriFab and Saporin; (**b**) Targeted delivery of Dig-Saporin with IgG-derived (two antigen binding sites + two Dig-binding sites) or Fab-derived (one antigen binding site + two Dig-binding sites) bsAbs of the same targeting specificity indicates that TriFabs have at least the same specificity and delivery potential as other bsAb Formats (monovalent cell surface targeting with LeY specificity is less potent than bivalent (avidity-enhanced) targeting).

TriFab-mediated targeted delivery of large molecules was demonstrated with digoxigenin-coupled saporin. Saporin is a plant-derived ribosome inactivating protein which becomes cytotoxic upon binding to and uptake into cells. By itself, however, saporin does not possess a cell binding functionality [21]. Because of that, only targeted delivery of saporin to and into cells generates cytotoxicity. Figure 3a shows that TriFabs (left panel) can be applied to specifically target Saporin to antigen expressing cells. Application of LeY-Dig binding TriFabs and Dig-saporin efficiently kills LeY expressing MCF7 cells. In contrast, Dig-Saporin by itself or coupled to TriFabs that recognize CD33 instead of LeY do not induce cytotoxicity in CD33 negative MCF7. Biotinylated saporin becomes specifically delivered to target cells in the same manner by Bio-binding TriFabs, however with somewhat reduced potency compared to Dig-Saporin (suggesting that the attached hapten may modify payload potency). A comparison with targeted delivery of Dig-Saporin by previously described IgG-derived (2 + 2) bsAbs (two binding entities for each target, [11]), or with Fab-derived fusion proteins (one cell surface binding entity) revealed that TriFabs retained at the same payload delivery potency than Fc-containing (bivalent target addressing) bsAbs and appear to have better potency compared to Fab-derived bsAbs that bind the LeY antigen in a monovalent manner (Figure 3).

## 3. Experimental Section

### 3.1. Expression of TriFabs

TriFabs were produced by co-transfection of three expression plasmids [11]. One plasmid encodes the L-chains of desired antibodies, the other two plasmids encode separate modified H chains. The positions of mutations and alterations in these H-chains are defined by the Kabat numbering convention [8]. These two H-chains contain linker peptides without disulphides instead of the hinge region, and VHcys44 or VLcys100 domains fused to CH3-domains with disulphide-stabilized knobs or holes heterodimer (T366W + T366S, L368A, Y407V; + S354C-Y349C disulphide) respectively. The components become expressed by CMV promoter driven transcription in HEK293 suspension cells that are grown at 37 °C in a humidified 8% CO_2_ environment. Seven days after transfection, culture supernatants that contain the secreted assembled antibody derivatives are sterile filtered and either immediately subjected to purification (Figure 1c), or stored frozen at −80 °C (thawed at room temperature prior to purification).

### 3.2. Purification of TriFabs

Hi Trap Kappa-select (GE Healthcare, Amersham Place, Little Chalfont, UK) is applied as first purification step as the molecules that we generated did not bind to protein A (see supplemental data). After loading supernatants to the column (left peak in Figure 1C) TriFabs were eluted with 100 mM Glycine-buffer (pH 2.5), subsequently adjusted to pH 6.0–7.5 with 1M Tris (pH 9.0). Subsequently, homogenous TriFab preparations are obtained by applying size exclusion chromatography (SEC, Superdex200 HiLoad 16/60, GE Healthcare) equilibrated with 20 mM histidine, 140 mM NaCl, at pH 6.0 on an Aekta Avant (GE Healthcare) as previously described for IgG-derived bispecific antibodies [11]. Yields were between 3–20 mg TriFab/L (2LeY-1Dig = 3.0 mg/L, 2Dig-1LeY = 5.7 mg/L, 2CD33-1Dig = 20.3 mg/L, 2Dig-1CD33 = 6.9 mg/L 2GPC3-1Dig = 3.5 mg/L, 2Dig-1GPC3 = 8.3 mg/L).

### 3.3. Characterization of TriFabs

FACS analyses were applied to assess specific binding of TriFabs to cell surface antigens as well as targeted delivery of small compounds. Therefore, cells were exposed to hapten-binding TriFabs followed by incubation with haptenylated fluorophores [11,13,14]. Specific binding is indicated by detection of TriFab-mediated fluorophore accumulation on cell. To analyze TriFab mediated targeted delivery of protein toxins, cells which either do or do not express the cognate antigen on their surface cultured in 96 well plates are exposed to TriFab-Toxin complexes for 48 to 72 h. Subsequently, DNA synthesis is determined by BrdU incorporation assays after 48 h. Affinities of recombinant TriFabs were determined by Surface Plasmon Resonance measurements as previously described [11].

### 3.4. Stability Analyses

Thermal stability was assessed using an Optim1000 instrument (Avacta Analytical Inc., Thorp Arch Estate, Wetherby, UK) recording light scattering and tryptophan fluorescence simultaneously while heating samples with a constant heat rate. Samples were prepared at 0.3–1 mg/mL in 20 mM histidine, 140 mM NaCl, pH 6.0 and transferred to a 9 µL multi-cuvette array and heated from 30 to 90 °C at a constant rate of 0.1 °C/min. The intensity of scattered light and the fluorescence emission spectra was recorded after excitation with a 266 nm laser providing a data point approximately every 0.6 °C. Light scattering intensities were plotted against temperature and aggregation onset temperature (Tagg) defined as the temperature at which the scattered light intensity begins to increase. For the unfolding readout, the ratio of the fluorescence intensities at 350 and 330 nm were plotted as a metric for the shift in peak position against the temperature. Denaturation temperature (Tm) is defined as the curve inflection point (Figure S2).

## 4. Conclusions

TriFabs are shaped like IgGs, composed of antibody derived domains, and of sufficient size (150 kDa) to avoid renal clearance. In contrast to IgG’s, they lack CH2 domains. These domains, in particular residues and structures at the CH2-CH3 interface, are important for binding of IgGs to Fc-interacting molecules including Fc-receptors and protein A (PDB:1L6X, [22,23]). Alternative interactions of protein A with VH (VH3) domains have also been described (PDB:1DEE, [24]), but those do not enable protein A binding of our molecules. In consequence, our TriFabs do not bind to protein A (see supplemental data).

Presence of a functional CH2 and of an intact CH2-CH3 interface region is also required to bind to the neonatal Fc receptor (FcRn, [25,26]). Lack of CH2 prevents interaction with FcRn and hence, without that, TriFabs will not undergo FcRn mediated recycling. Because of that, it is very likely that TriFabs will have pharmacokinetic properties similar to IgG derivatives that are devoid of FcRn binding sites [25,26], which needs to be confirmed in animal studies.

Removal of CH2 affects not only the pharmacokinetics of TriFabs but renders them also deficient in other Fc functionalities. This includes lack of induction of antibody-dependent cell-mediated cytotoxicity (ADCC) which is triggered by binding of Fc-Receptors (FcgRIII), involving CH2. ADCC is an important contributor to therapeutic efficacy of antibody therapies, in particular for protection from viral infections or for ADCC mediated elimination of tumor cells. Obviously, such therapeutic approaches in virology or oncology that have ADCC induction (or other Fc mediated functionalities) as major efficacy contributors cannot be met by a CH2-deficient TriFab. On the other hand, inability to trigger ADCC can be desired if one aims at antibody-mediated neutralization or depletion approaches (for example removal of angiogenic ligands or removal of inflammatory stimuli) while avoiding direct and potentially damaging cellular effects.

Lack of ADCC competence is of minor concern for bsAb mediated targeted delivery of cytotoxic payloads into cells, exemplarily shown in Figure 3. This principle (ADCs and ADC like molecules) requires effective internalization of antibody-payload complexes following target cell binding and is hence rather incompatible with ADCC (IgG needs to be surface accessible to trigger ADCC). In contrast to most ADCs, tumor-targeting hapten-binding TriFabs are defined entities that have cytotoxic payloads coupled to the stem-Fv in a position- and stoichiometry-defined manner. Such TriFabs are therefore well suited for payload targeting approaches.

Many different antibody formats have been generated since the take-off of the bispecific antibody field and its proven applicability for diagnosis and therapy [1,2,3,4,5,6,7,9,27,28,29,30]. This includes small Fv-derived entities with short serum half-life due to renal filtration (such as BiTEs, [25]), as well as large Fc containing molecules with extended serum half lifes [11,13,14,15]. The majority of bsAb formats that have been applied so far (and that are in clinical development) are composed of 1 + 1 or 2 + 2 formats, *i.e.*, possess one binding site for each different antigen or two binding sites per antigen [1,2,3,5,6,9,14,15,28]. Some selected examples for previously published 2 + 1 formats (similar to TriFabs with two binding entities for one and one entity for another antigen) have been generated by the dock&lock method [30], or as knob-into-hole IgGs fused to disulphide-stabilized Fv’s [4]. All these 2 + 1 formats differ in “binding geometry”, *i.e.*, positioning and special orientation/distance of the binding modules to each other. One additional advantage of the TriFab format over other knob-into-hole containing bsAbs is that the fusion of additional heterodomerization promoting modules (VH and VL) to the modified CH3 domains results in a “super-heterodimerization” entity. Desired heterodimerization of the stem region is thereby promoted by two distinct interactions, each of which by itself being already sufficient to drive heterodimerization. CH3 knob-hole interactions by themselves are sufficient for heterodimerization, the VH and VL domains of the stem region (also independently sufficient) provide additional contributions, and the generated stem region is further stabilized by an interchain disulphide between VH and VL.

Valency, orientation or distance between binding modules are parameters that influence the functionality of bispecific antibodies, dependent on targets to be addressed and functionalities to be achieved. Because of that, there is not one “optimal format” for bsAbs. Instead, different formats may need to be applied for different applications. For example, bivalency of binding to cell surface antigens may be desired to achieve preferential (avidity mediated) binding to cells with abundant cell surface target expression. On the other hand, bivalent engagement of cell surface targets such as receptors may (dependent on the addressed targets) also change their internalization, and thereby either promote or attenuate uptake of bsAbs and of attached payloads. Other indications such as “bridging approaches” aim at generating tight connections between targets or target cells while other applications need rather independent separate binding events (e.g., to inactivate two soluble ligands or for targeted payload delivery).

Regarding cell targeting approaches, the binding geometry of TriFabs with two normal Fab arms and one interspersed stem-Fv mediates efficient (and avidity enabled) binding of the Fab arms. Monovalent binding of the interspersed Fv may also be unrestricted for some accessible and/or flexible cell surface antigens (carbohydrates/glycans may be particularly suited as paratope 2 antigens). However, paratope 2 binding may also be sterically hindered, depending on the target antigen and epitope in particular for large and/or complex antigens. For example, the antigen 2 may need to “squeeze” between the paratope 1 binding Fab arms, which would affect the on-rate in a similar manner as described in [4]. Such reduced binding affinity to paratope 2 (monovalent and potentially sterically compromised) may, in some cases of cell surface targeting approaches, be compensated by the bispecific binding principle: the unrestricted bivalent Fab arms keep the TriFab in place and prevent its dissociation from cells. This, in turn, provides additional time for the interspersed Fv to bind (or re-bind in case of dissociation due to monovalency), compensating for a “bad” on-rate of the interspersed binding module. Thus, the bsAb principle can compensate potential affinity deficits of paratope 2 binders on cell surfaces, provided the bsAb geometry permits simultaneous binding of both paratopes. Simultaneous binding of two antigens may be applied to address “close proximity” requirements, which are necessary for inducing cell to cell contacts, e.g., in cancer immune-therapy.

In conclusion, TriFabs can be applied to simultaneously address or crosslink accessible target antigens, for imaging, or for targeted (or pre-targeted) delivery of small and large payloads to tumor cells.

## Supplementary Materials

Supplementary materials can be found at http://www.mdpi.com/1422-0067/16/11/26037/s1.
