# Supplementary material for: TriFabs—Trivalent IgG-Shaped Bispecific Antibody Derivatives: Design, Generation, Characterization and Application for Targeted Payload Delivery
Source: Int J Mol Sci. 2015 Nov 17;16(11):27497–507. doi: 10.3390/ijms161126037 (PMC4661895; doi:10.3390/ijms161126037)
Supplement: Supplementary file 1 [file ijms-16-26037-s001.pdf]

Supplemental Materials: TriFabs—Trivalent IgG-Shaped Bispecific Antibody Derivatives: Design, Generation, Characterization and Application for Targeted Payload Delivery

Klaus Mayer, Anna-Lena Baumann, Michael Grote, Stefan Seeber, Hubert Kettenberger, Sebastian Breuer, Tobias Killian, Wolfgang Schäfer and Ulrich Brinkmann

Purification of TriFabs

TriFabs are purified from cell culture supernatants by affinity and size exclusion chromatography. Due to lack of CH2 domains, TriFabs do not bind to Protein A (Figure S1c: TriFabs pass a Protein A column (left panel) while CH2 containing bsAbs [10] bind to and can be eluted from Protein A (right panel)). TriFabs are therefore purified by Protein L affinity chromatography followed by size exclusion chromatography (Figure S1). Hi Trap Kappa-select (GE Healthcare) is applied as first purification step, TriFabs eluted with 100 mM Glycine-buffer (pH 2.5) and adjusted to pH 6.0–7.5 with 1M Tris (pH 9.0) are subsequently subjected to SEC on a Superdex200 HiLoad 26/60 (GE Healthcare) equilibrated with 20 mM histidine, 140 mM NaCl, at pH 6.0 on an Aekta (GE Healthcare). Shaded boxes in Figure S1 (SEC profiles) indicate fractions containing properly folded TriFab. The composition and purity of TriFabs obtained by this simple 2-step procedure is shown in the SDS PAGE without (n.r.) and with (r.) sample reduction (right panel). (A) depicts SEC and SDS-PAGE of TriFabs containing the hapten-binding entity in the stem region; (B) of TriFabs with cell-binding entities in the stem region.

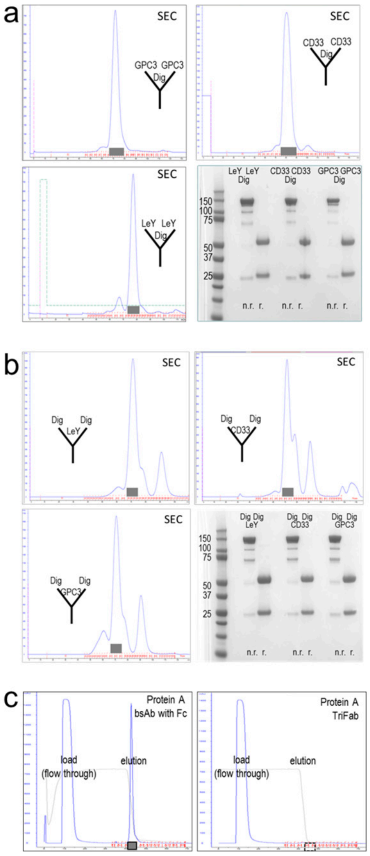

Figure S1. Purification of TriFabs. (**a**) TriFabs harboring hapten binding entities in their stem regions; (**b**) TriFabs harboring hapten cell targeting entities in their stem regions; (**c**) Fc containing bsAbs bind to protein A (**left** panel) but TriFabs do not bind protein A (**right** panel).

Thermal Stability of TriFabs

Thermal stability was assessed by recording light scattering and tryptophan fluorescence simultaneously while heating samples with a constant heat rate (see Experimental Section for sample preparation and experimental details). Samples were heated from 30 to 90 °C at a constant rate of 0.1 °C/min with continuous recording of the intensity of scattered light and the fluorescence emission spectra after excitation with a 266 nm laser. For the aggregation onset temperature readout, light scattering intensities were plotted against the temperature as shown in Figure S2a. The aggregation onset temperature (Tagg) is defined as the temperature at which the scattered light intensity begins to increase. For the unfolding readout, the ratio of the fluorescence intensities at 350 and 330 nm were plotted as a metric for the shift in peak position against the temperature as shown in Figure S2b. The denaturation temperature (Tm) is defined as the inflection point in these curves. Aggregation onset and denaturation curves of TriFabs that bind to different cell surface antigens and different haptens are shown in Figure S2: Figure S2a demonstrates aggregation onset temperature of TriFabs (scattered light intensity of an incident 266 nm laser during continuous heating in an Optim1000 instrument; aggregation onset temperature (Tagg) is defined as the temperature at which the scattered light intensity begins to increase). Figure S2b depicts denaturation of TriFabs (the ratio of the fluorescence intensities at 350 and 330 nm was recorded during the same experiment as in (Figure S2a); denaturation temperature (Tm) is defined as the inflection point in these curves). The results of these analyses are summarized in Table 1. These indicate that TriFabs are quite stable, with aggregation onset temperatures between 51 and 61 °C and denaturation temperatures between 58 and 66 °C. The only (format independent!) variation in stability that we observed was that Dig-binder containing TriFabs tolerated even higher melting temperatures (>60 °C) than Bio-binders (Table 1).

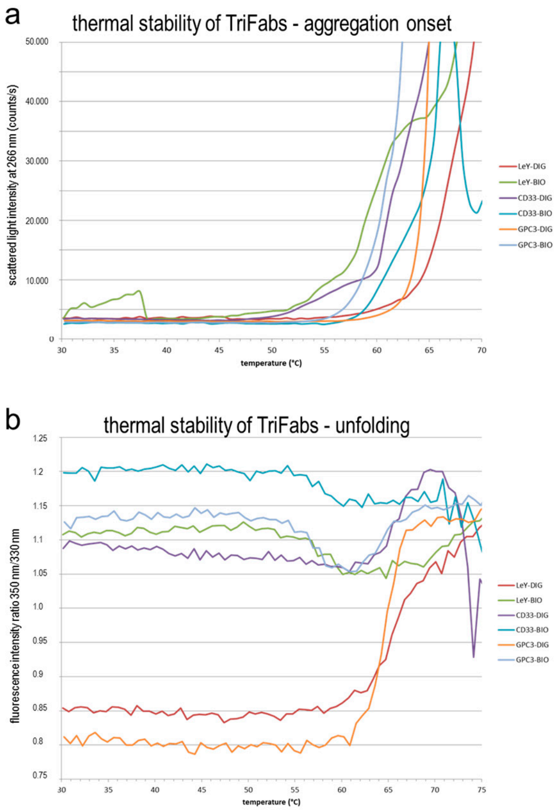

Figure S2. Stability of TriFabs as determined in thermal denaturation experiments assessing aggregation onset (**a**) or unfolding (**b**).
